# The Use of Corneoscleral Grafts to Maintain Tectonic Stability in Severe Keratolysis

**DOI:** 10.3390/vision7030062

**Published:** 2023-09-21

**Authors:** Lennart M. Hartmann, Hans-Juergen Buchwald, Carolin Elhardt, Efstathios Vounotrypidis, Armin Wolf, Christian M. Wertheimer

**Affiliations:** Department of Ophthalmology, Ulm University, Prittwitzstraße 43, 89075 Ulm, Germany; hans-juergen.buchwald@uniklinik-ulm.de (H.-J.B.); carolin.elhardt@uniklinik-ulm.de (C.E.); efstathios.vounotrypidis@uniklinik-ulm.de (E.V.); armin.wolf@uniklinik-ulm.de (A.W.); christian.wertheimer@uniklinik-ulm.de (C.M.W.)

**Keywords:** corneoscleral grafts, keratoplasty, keratolysis, tectonic stability

## Abstract

Severe corneal ulcerations, causing major keratolysis with large perforation of the cornea or extending to the limbal region, are an ophthalmic emergency. In these cases, a larger corneoscleral graft can be transplanted to restore tectonic integrity, alleviate pain, save vision, and prevent loss of the eye. Chart review of 34 patients with a corneoscleral graft ≥9.5 mm was conducted. Primary endpoints of the study were tectonic stability defined as no need for another keratoplasty or enucleation. In addition, visual acuity, postoperative complications, and secondary procedures were analyzed. In total, 12 patients (35%) were female. The mean age at transplantation was 65 ± 19 years. The underlying disease was a perforated infectious corneal ulcer in 30 cases (88%). Mean follow up was 675 ± 789 days. Tectonic stability at the end of the follow-up was maintained with a probability of 56% in a Kaplan–Meier analysis. Another penetrating keratoplasty was necessary in six cases (17%) and enucleation in five cases (15%). A corneoscleral transplant remains a viable treatment option to prevent enucleation in severe keratolysis. In our study, this was possible in about half of the cases. Postoperative complications, secondary surgeries, and markedly reduced visual acuity put the advantages into perspective.

## 1. Introduction

Severe corneal ulcers, especially in the setting of microbial keratitis, that cause extensive keratolysis with perforation of more than a portion of the cornea or extending to or beyond the limbus, are ophthalmic emergencies [1]. In these cases, a larger corneoscleral graft can be transplanted to restore tectonic integrity, relieve pain, prevent complications, save vision, and prevent loss of the eye [2].

Even if the eye would otherwise have been lost, larger grafts carry a high potential for complications [3]. In addition to the complex surgical nature of severely altered corneal tissue, biological factors play an important role and underscore the difficulty in managing these cases [4]. With a large graft, the corneal limbus is removed or damaged, resulting in persistent epithelial defects and ingrowth of conjunctival tissue and vessels. As graft size increases, so does the rate of rejection due to contact with the highly vascularized scleral tissue and the resulting host immune response. Another problem is the recurrence of the underlying disease, such as infection [5].

Although corneoscleral transplantation is a rare procedure and has known disadvantages, it can rescue end-stage ocular disease in some patients and is used for this purpose [6]. In the present study, patients with various clinical entities requiring emergency corneoscleral transplantation were evaluated to determine both its utility in maintaining the tectonic stability of the globe and the postoperative clinical course, including complications, secondary procedures, and visual acuity development.

## 2. Materials and Methods

### 2.1. Study Design and Subjects

This is a retrospective, longitudinal single-center study of 34 patients who received a corneoscleral grafts of ≥9.5 mm for perforated corneal ulcers at the Department of Ophthalmology between 1 January 2010 and 9 January 2022. This retrospective analysis was approved by the ethics committee of the University of Ulm (Approval ID: 178/21) and adhered to the tenets of the Declaration of Helsinki. Data were extracted from charts. Thirty-four patients were identified with perforated corneal ulcers so severe and extensive that only a corneoscleral graft ≥ 9.5 mm could salvage the eye. Graft size ranged from 9.5 to 14 mm with a mean size of 11.6 ± 1.4 mm. Of these patients, 12 (35%) were female. The mean age at transplantation was 65 ± 19 years. The underlying disease was infectious in 30 cases (88%). One case was Acanthamoeba (3%), three cases were fungal (9%), and five cases were herpes simplex (15%). Five non-infectious causes (15%) included chemical burns, exposure keratopathy, and neurotrophic keratopathy. The mean graft size was 10.8 ± 1.2 mm.

### 2.2. Surgical Procedure

Written informed consent was obtained for the procedure and to ensure understanding of the poor prognosis and regular follow-up. Surgery was performed under general anesthesia by six different experienced surgeons. The planned step-by-step procedure was as follows but was regularly modified for individual approach and management of complications: (1) Sterile draping and disinfection with povidone iodine, taking into account any perforation. (2) A surgical compass was used to determine the diameter of the patient’s corneoscleral tissue to be removed. (3) The graft was marked with a corneal radial keratotomy marker. (4) The donor graft was prepared prior to any surgery on the recipient eye. (5) The diameter of the donor graft was 0.5-1 mm larger than the diameter of the tissue to be removed from the recipient. (6) The donor graft was placed on a plastic block, taking care not to desiccate the endothelium. (7) A central corneoscleral button was sutured. (8) The corneoscleral button was placed in surgical viscoelastic. (9) A cell culture medium swab was taken. (10) The conjunctiva was opened and moved away from the limbal region to expose 360° of the paralimbal sclera. (11) Bleeding during dissection was stopped with an electrocautery device. (12) The recipient was marked with a corneal radial keratotomy marker. (13) A Flieringa scleral fixation ring was sutured to the sclera at 4 equidistant points with 7-0 silk sutures. (14) The recipient eye was opened by paracentesis or entered through a perforation site and a viscosurgical device was injected into the anterior chamber. (15) Removal of the recipient corneoscleral tissue requires an individualized approach. The following considerations were made on a case-by-case basis to remove lytic tissue while maintaining the best possible function: (a) A round trephine was always used, often in combination with an ophthalmic crescent blade and round corneoscleral scissors. (b) To preserve the iridocorneal angle structures and avoid choroidal exposure, the sclera was not trephined in full thickness. A scleral lamella was removed manually with a crescent blade in a planned thickness of approximately 400 µm to create a scleral ledge. (c) The graft was sometimes decentered to preserve the limbal region. (d) Electrocautery is often necessary but causes tissue contraction. (16) The donor graft was attached with four cardinal sutures and secured with multiple 8-0 to 10-0 nylon sutures (24–40 sutures total). (17) All suture knots were buried. (18) The Flieringa scleral fixation ring and viscoelastic were removed. (19) Sutures were replaced if graft–host junction leakage was detected. (20) Balanced salt solution and intracameral cefuroxime were injected. (21) The conjunctiva was approximated to the limbus with 9.0 absorbable sutures. (22) The eye was patched. Postoperative topical therapy varied depending on the underlying disease, but usually included prednisolone and antibiotics. Lubrication was also prescribed. Due to the size of the grafts, the transplantation of scleral tissue and the resulting high risk of graft rejection, oral immunosuppression was prescribed in all cases. Postoperative weight-adjusted prednisolone and/or mycophenolate mofetil 1 g was administered orally twice daily.

### 2.3. Study Endpoints

The primary endpoint of the study was tectonic stability. Maintaining tectonic stability after the initial surgery was further defined as not requiring additional penetrating keratoplasty or enucleation. If no additional surgery was required to maintain tectonic stability, the initial surgery was defined as successful. Secondary endpoints were final visual acuity excluding the 5 enucleations and complications and secondary surgeries.

### 2.4. Follow-Up Examinations

Patients were regularly followed up in the outpatient clinic at appropriate intervals. Patients were readmitted to the inpatient clinic in case of complications or loss of tectonic stability. Medical history, intraocular pressure, corrected visual acuity, a comprehensive slit-lamp examination and the treatment plan were recorded at each follow-up visit. Grafts were evaluated for complications, such as re-infection, graft rejection, hypotony, or secondary glaucoma. Underlying conditions, both infectious and non-infectious, such as neurotrophic or exposure keratopathy were also treated as appropriate.

### 2.5. Statistical Analysis

Statistical analysis was performed using unpaired t-test for parametric data or the Mann–Whitney test for nonparametric data. Graft survival was analyzed via simple Kaplan–Meier survival analysis. This was performed using GraphPad Prism (version 9). Statistical significance was set at a *p*-value < 0.05.

## 3. Results

### 3.1. Tectonic Stability

**Figure 1:**
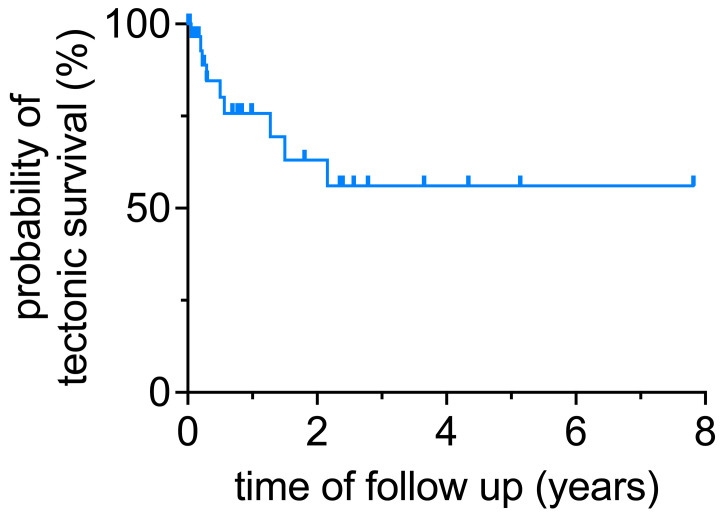
Kaplan–Meier estimators were calculated and plotted for tectonic stability as defined by the need for additional penetrating keratoplasty or enucleation. At the end of the follow-up, the probability of tectonic stability was 56%.

**Figure 2:**
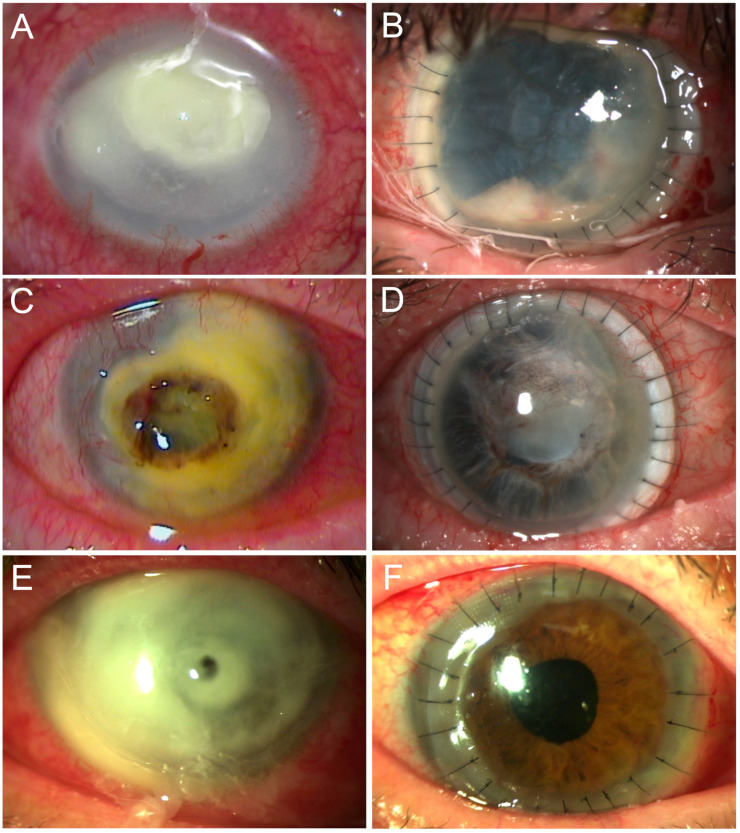
Case examples from this study are shown. A preoperative fungal ulcer caused by Fusarium (**A**), 17 months after corneoscleral transplantation with recurrent anterior segment infection before enucleation (**B**). A patient with a herpetic ulcer with keratolysis in the whole limbal area and a large central perforation preoperatively (**C**) and five months after successful corneoscleral transplantation, closed epithelium and mature cataract (**D**). A patient with a large bacterial ulcer with central perforation preoperatively (**E**) and 5 days after successful corneoscleral transplantation (**F**).

The primary endpoint of the study was tectonic stability, defined by the need for additional penetrating keratoplasty or enucleation. Using this definition, the proportion of eyes with a probability of tectonic stability at the end of follow-up was 56% as estimated via Kaplan–Meier analysis (Figure 1). Reasons for the six re-keratoplasties included recurrent infection in three cases, graft rejection in two cases, and traumatic dehiscence of the graft in one case. In five cases (14%), tectonic stability could not be maintained, and enucleation was necessary. Reasons for enucleation were refractory infection in three cases and expulsive suprachoroidal hemorrhage in one case. Two of these enucleations were performed in eyes that had already been regrafted (Figure 2).

### 3.2. Follow-Up and Complications

Concurrent with the procedure, all patients received intensive topical treatment tailored to their underlying disease and needs. All patients were treated with topical prednisolone 1%. In addition, systemic immunosuppressants, antivirals, and antibiotics were used, as well as contact lenses when appropriate for non-infectious epithelial defects. Because corneoscleral transplantation can be a difficult procedure and is often performed in very severe cases, the risk of postoperative complications is significant. During our mean follow-up of 675 ± 789 days, several complications were observed. The most common complication was epithelial defect in 13 cases (38%), followed by postoperative intraocular pressure decompensation in 12 cases (35%), and recurrent infection in 9 cases (26%). Postoperative hypotony occurred in five cases (15%). Oral immunosuppressive therapy was used as recommended in 22 cases (65%) and rejection occurred in 10 cases (29%). Graft size seemed to play an important role as the graft size of patients with graft rejection was significantly larger than the graft size of patients without rejection in our cohort (*p* = 0.0005).

### 3.3. Visual Acuity

**Figure 3:**
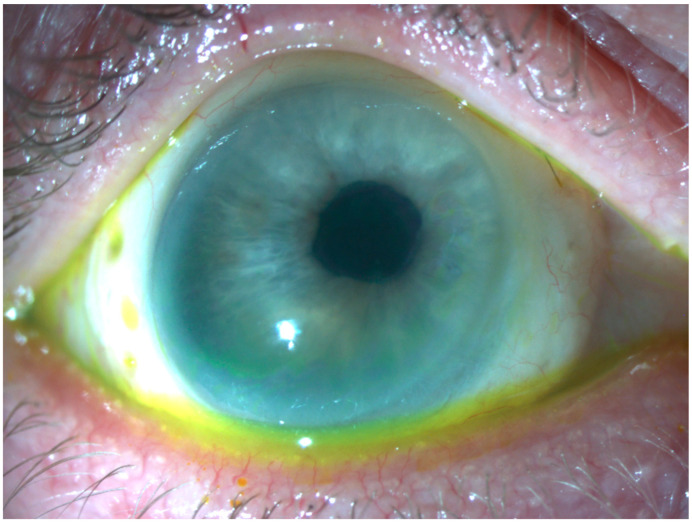
Although corrected visual acuity in our cases was generally low, there was one notable case. Eight years after a corneoscleral graft for a perforated corneal ulcer (fusarium), the graft remained clear, and a visual acuity of 0.3 logMAR was observed.

Overall, 18 patients (62%) had a final visual acuity of hand movements or worse, 7 (24%) had a final visual acuity of counting fingers to 1.0 logMAR, and 5 (17%) had a final visual acuity of <1.0 logMAR. In general, visual acuity was poor, but there was one notable exception among our patients. The patient had a transplantation due to a severe fusarium-related perforated corneal ulcer. The graft was completely clear with a visual acuity of 0.3 logMAR after 8 years without systemic immunosuppression (Figure 3).

### 3.4. Secondary Surgical Interventions

When postoperative problems could not be managed by appropriate adjustment of local therapy, surgery was performed (Table 1). Repeat grafting was required in six cases (17%). In addition, temporary partial tarsorrhaphy was required in six cases, and amniotic membrane grafting was required in eight cases due to persistent epithelial defect. One botulinum toxin-induced ptosis and one eyelid correction were performed for the same reason. Two cases required re-suturing due to suture loosening. One case developed a postoperative rhegmatogenous retinal detachment, which was treated via a pars plana vitrectomy. In five cases (14%) tectonic stability could not be maintained and enucleation was required.

## 4. Discussion

The treatment of extensive corneal ulcers causing severe keratolysis with perforation of more than a portion of the cornea and extending to or beyond the limbus remains a challenge. In these cases, a corneoscleral transplantation is a treatment option to preserve the eye. In our study, this was possible in approximately half of the cases. Postoperative complications, secondary surgeries, and significantly reduced visual acuity put the advantages into perspective.

There are few studies that comment on the development of visual acuity, and the general impression is that tectonic stability is what can be expected from the procedure [7]. Some authors also suggest that optical penetrating re-keratoplasty during an inflammation-free interval may be a valid option to improve visual acuity [8]. This is consistent with our study, as only five patients achieved visual acuity better than 1.0 logMAR. We hypothesized that concomitant systemic immunosuppression would have a positive effect on the final visual acuity. However, there was no significant difference in the final visual acuity between patients who received oral immunosuppression and those who did not (*p* = 0.45). Regarding tectonic stability in severe infections, in another study of 60 large grafts, 7 eyes underwent regrafting, 7 eyes developed phthisis bulbi, and a tectonic outcome was achieved in 80% of cases, which was slightly higher than in our study [9]. In another study with 14 grafts, tectonic stability was observed in all included patients. However, only ulcers with a rheumatologic etiology were included, whereas the vast majority of ulcers included in our study were of infectious origin, which may contribute to different results [10]. The differences in clinical outcomes between infectious and sterile perforated ulcers have also been demonstrated elsewhere [11]. Regarding the rate of re-keratoplasty, our results were comparable to those in the previously published literature. In one study, the rate of re-keratoplasty was 17%, which is identical to the 17% we found [7]. In our cohort, different causes may have contributed to the determination of success or failure of globe integrity. As mentioned above, the pathogenesis of the ulcer plays an important role for the clinical outcome. In two out of nine cases, globe integrity was lost due to recurrent mycotic ulcer, although only 3 of 34 patients suffered from mycotic ulcer as a result of keratoplasty.

Elevated intraocular pressure is a common complication after corneoscleral transplantation, so intraocular pressure must be monitored regularly as it can lead to glaucoma. The postoperative use of steroids can also lead to increased intraocular pressure. The current report and previous studies are in agreement regarding the increase in intraocular pressure. In our study, 34% of patients suffered from increased pressure, while another group reported 57% [3]. In particular, large corneoscleral grafts remove the entire trabecular meshwork and Schlemm’s canal, so theoretically an increase in intraocular pressure would be expected in all eyes, but this was not observed in the present and previous studies. It is still unclear how aqueous humor outflow is achieved in eyes with corneoscleral grafts. Because of the major surgical trauma, other outflow pathways besides the transplanted trabecular apparatus would be possible. Preservation of a scleral ledge extending to the cornea or sclerocorneal dissection and angle-supporting sutures to preserve the angle and its function have been performed in previous reports with good results [12]. Scleral ledge preservation was also used in all of our patients.

In general, it is difficult to comment on the average survival of corneoscleral grafts due to the lack of precise information in the published studies and the high variance of the study population between the different studies. In addition, the definition of tectonic stability also varies between studies. The mean survival of corneoscleral grafts ranges from 24 months [12] to 20 months [5]. In another study, the mean time between corneoscleral grafting and loss of eyes in those cases where tectonic stability could not be maintained was 17 months [3].

There are reports describing both long-term graft survival. For example, a corneoscleral graft was used for 18 years with excellent visual results [13]. Another study reported a case where visual acuity at the end of follow-up was 0.1 logMAR in addition to long-term graft survival [5]. In our study, a patient with a large corneoscleral graft was observed to have a visual acuity of 0.3 logMAR after a follow-up period of 8 years presenting with a perfectly clear graft. To understand the pathology leading to the low retention rates in all other patients, it would be of great interest to shed light on what contributes to graft retention in these rare cases. 

The most frequently reported postoperative complication in our study was a persistent epithelial defect. This may be due to the fact that most grafts were larger than 10 mm, and, therefore, some allogeneic limbal tissue was transplanted and later rejected, or that the recipient’s limbus was altered either by the surgery or by the severe inflammatory conditions prior to surgery. Donor keratolimbal tissue has been shown to promote corneal epithelial healing in severe ocular surface disease [14]. Immunosuppression, quiescent conditions, and human leukocyte antigen matching have been described as prerequisites for this benefit [15]. Many of the ulcers treated in this study were due to severe infections, where recurrent infections and persistent inflammatory processes may limit limbal function. The use of topical medications may also have led to limbal insufficiency [16]. Solving this problem may significantly improve graft survival in the future.

The immune privilege of the cornea guarantees the high graft survival rates observed in low-risk keratoplasty [17]. Unfortunately, this privilege is lost in corneoscleral grafts due to contact with highly vascularized scleral or limbal tissue. Immunosuppression may improve graft survival and clarity. The evidence for postoperative immunosuppression is still limited. Forty-four percent of surgeons in the United Kingdom administer prednisolone perorally after surgery for high-risk keratoplasty [18]. Mycophenolate mofetil is the standard therapy of care in our clinic and has been reported to reduce the rate of rejection three years after surgery but has no effect on graft failure [19]. Cyclosporine A and tacrolimus have also been described with mixed results and there is no clear recommendation for their use, partly because the scientific evidence is limited [20]. It should be noted that systemic immunosuppressants may be associated with side effects, for which patients should be monitored. Another option is to match donor and recipient human leukocyte antigens [21]. This was not possible in our study because all procedures were emergencies and the waiting time for a matched transplant is significantly longer than for an unmatched one. Newer immunomodulatory approaches are currently under investigation and may prevent graft failure in this high-risk group with a better side effect profile [20].

Lamellar keratoplasty techniques, such as large deep anterior lamellar keratoplasty, may be an interesting technique for large keratolysis with descemetocele, for example in severe herpetic keratitis. One case report showed a healthy graft and relatively clear interfaces 6 months after surgery [22]. Another group reported seven cases of large-diameter deep anterior lamellar keratoplasty for deep vascularized corneas with severe peripheral thinning after Acanthamoeba keratitis with good results [23]. The technique has the potential advantage of preserving certain structures, such as the endothelium, which may lead to lower rejection and complication rates. A potential disadvantage is the theoretically higher risk of not eradicating infectious agents.

Limitations of the study are related to the rarity of the procedure and the disease treated. These include the retrospective nature of this study and the lack of a control group. Data had to be obtained from clinical records. Surgery was performed by different surgeons with different follow-up times. Future studies with larger numbers of patients are needed to further specify when corneoscleral transplantation should be performed and what strategies should be used to allow long-term graft survival and, thus, preserve the integrity of the globe.

## 5. Conclusions

Severe corneal ulceration is a serious threat to visual acuity and often results in loss of tectonic stability and ultimately of the eye. Corneoscleral grafts represent a treatment option to achieve tectonic stability and prevent enucleation in these cases. In this study with a large cohort of 34 patients, the integrity of the otherwise lost globe was preserved in more than half of the cases. Prognosis of visual acuity is guarded, except for notable exceptions, and is not the primary goal of treatment.

## Figures and Tables

**Table 1:** Patient cases, etiology, secondary procedures, and outcome.

Cases n (%)	Etiology	Secondary Procedures Until Stability Was Lost (n)	Outcome (Stability Achieved/Re-Keratoplasty/Enucleation)
1 (3%)	Acanthamoeba	Amniotic membrane transplantation (1)	(1/none/none)
20 (59%)	Bacterial	Lentectomy (1), amniotic membrane transplantation (6), tarsorrhaphy (3), other eyelid surgery (1), Botulinum induced ptosis (1), re-suturing (1)	(16/2/2)
3 (9%)	Fungal	Amniotic membrane transplantation (2), tarsorrhaphy (1)	(1/1/1)
5 (15%)	Herpes Simplex	Amniotic membrane transplantation (1), tarsorrhaphy (1)	(5/none/none)
5 (15%)	Non-infectious	Pars plana vitrectomy (1), tarsorrhaphy (1), re-suturing (1)	(2/1/2)

## Data Availability

The data presented in this study are available on request from the corresponding author. The data are not publicly available due to ethical restrictions.
